# Estimating UMI-Tag count: UMI-TnSeq protocol for identifying fitness-relevant genes in the PCB degrader *Acidovorax* sp. KKS102

**DOI:** 10.1093/dnares/dsag017

**Published:** 2026-10-30

**Authors:** Yoshiyuki Ohtsubo, Yutarou Hemmi, Shiho Abe, Shouta Nonoyama, Kouhei Kishida, Yuji Nagata

**Affiliations:** Department of Molecular and Chemical Life Sciences, Graduate School of Life Sciences, Tohoku University, Sendai 980-8577, Japan; Department of Molecular and Chemical Life Sciences, Graduate School of Life Sciences, Tohoku University, Sendai 980-8577, Japan; Department of Molecular and Chemical Life Sciences, Graduate School of Life Sciences, Tohoku University, Sendai 980-8577, Japan; Department of Molecular and Chemical Life Sciences, Graduate School of Life Sciences, Tohoku University, Sendai 980-8577, Japan; Department of Molecular and Chemical Life Sciences, Graduate School of Life Sciences, Tohoku University, Sendai 980-8577, Japan; Department of Molecular and Chemical Life Sciences, Graduate School of Life Sciences, Tohoku University, Sendai 980-8577, Japan

**Keywords:** UMI, GIIF, PCBs

## Abstract

We developed a novel UMI-TnSeq (unique molecular identifier TnSeq) protocol to enhance the accuracy of quantifying the relative abundance of Tn mutants within a library. This improved approach includes custom-designed Tn-cassettes embedded with Illumina sequencing-compatible sequences and a unique adapter ligation protocol using reverse transcriptase to incorporate UMIs efficiently. Applying this refined UMI-TnSeq to the PCB/biphenyl-degrading *Acidovorax* sp. strain SA17 enabled us to identify genetic alterations that increased relative abundance in biphenyl media, including disruption of *relA* and transposon insertions predicted to increase the expression of a gene cluster encoding benzoate transporters. In addition, we introduced 2 novel metrics: the Gini coefficient and the “N-estimate.” The Gini coefficient tracks selection progression by measuring shifts toward fitter mutants, while the N-estimate, calculated via model parameter fitting, evaluates UMI diversity and enables calculation of the “Detection Completeness Value.“ These mathematical improvements are broadly applicable and beneficial for other UMI-based studies.

## Teaser

We established a new UMI-TnSeq protocol for the assessment of Tn mutant library composition with improved quantitative accuracy. To enhance the utility of UMI-TnSeq, we introduced 2 key metrics: the Gini coefficient, which quantifies selection progression by tracking shifts toward fitter mutants, and the N-estimate, which evaluates UMI diversity and enables the calculation of the Detection Completeness Value. The application of this method to PCB/biphenyl-degrading bacteria demonstrated its effectiveness, identifying different genomic mutations increasing relative abundance in biphenyl-containing media. The UMI-TnSeq protocol, along with a series of Tn cassettes featuring different antibiotic resistance markers, would be beneficial for studies aimed at identifying fitness-related genes in bacteria under specific environmental conditions.

## 1. Introduction

Transposon (Tn) mutagenesis has been widely used as a genetic tool to identify genes associated with particular phenotypes in a wide variety of living organisms, including bacteria, plants, and animals. With the advent of next-generation sequencers (NGSs), various Tn-sequencing techniques have been developed, including TnSeq^1,2^, HITS,^3^ INSeq,^4^ TraDIS,^5^ and RB-seq.^6^ (see reviews.^7,8^) These techniques simultaneously identify each genomic location of the Tn insertion site. They have been used not only to identify essential genes but also genes related to fitness under different growth conditions.

Although these TnSeq techniques have been used for quantitative analysis of Tn mutant libraries, they suffer from an innate problem of quantitative accuracy. When identical NGS reads are obtained, there is no way to discriminate whether they are derived from different DNA molecules or from a single DNA molecule that was amplified by PCR, resulting in multiple NGS reads. The key to solving this problem is the use of a unique molecular identifier (UMI), which has been proposed and used to circumvent quantitative issues in various NGS techniques,^2,9^ including metagenomic analysis of bacterial community compositions,^10^ ribosome protection assay,^11^ and others. The use of UMIs in TnSeq has also been developed and named UMI-TnSeq.^12^

With the aim of improving the UMI-tag incorporation protocol for UMI-TnSeq, we have been developing a series of methods for the ligation of UMI-containing adapter DNA to sheared DNA ends, as reported in our previous studies.^13–17^ We found that DNA shearing by sonication produces fragments with diverse chemical and physical structures at their 3′ termini^16^ contrary to a commonly held but inaccurate model.^18^ We devised an efficient method to repair these termini, producing blunt 3′ hydroxyl ends.^16^ The reverse transcriptase from Molony Murine Leukemia Virus (MMLV-RT) has strong tailing activity^14^ and can append 3 to 5 guanine nucleotides (Gs) to blunt 3′ hydroxyl termini in the presence of the specific enhancer deoxycytidine.^15^ We also established the CIS reaction by MMLV-RT, in which a single-strand DNA with 3′ consecutive Cs (called GAO for guide adaptor oligonucleotide) is incorporated into the G-tail by synthesizing the complementary strand of the GAO.^17^ Recently, we demonstrated that *Taq* polymerase can also catalyze the CIS reaction.^13^ Unlike MMLV-RT, which produces flap-end products, *Taq* polymerase results in nicked DNA due to its flap-end nuclease activity. The overall reaction efficiency is so high that GAO with a sequence of Ns that serves as UMI was incorporated into more than 95% of the DNA ends generated by sonication. In this study, we used this new method for ligating UMI-containing adaptors, which offers an advantage over the existing method using a combination of mariner transposon and MmeI,^12^ which limits sequence identification to only 13 to 14 bp of genomic DNA. Our method enables recovery of longer sequence reads downstream of insertion sites, improving mapping and allowing for more comprehensive analysis of insertion sites.

*Acidovorax* sp. KKS102 is a beta-proteobacterial strain capable of degrading biphenyl and polychlorinated biphenyls (PCBs). It carries a *bph* operon (Fig. 1a) encoding enzymes for PCB/biphenyl degradation on an integrative and conjugative element.^19^ The operon is transcribed from a promoter named *pE* promoter, which is under the control of the BphS repressor.^20^ This repression is alleviated in the presence of a biphenyl degradation intermediate.^21^ The *pE* promoter is also under catabolite control mediated by the BphQ activator.^22,23^ In our previous study, the *pE* promoter was replaced by a series of promoters with different transcriptional activities.^24^ We observed that as the transcriptional level increased, the biphenyl degradation activity reached a plateau. This observation suggested that at the plateau, factors other than the *bph* operon transcriptional level limit the degradation activity.

**Fig. 1.**
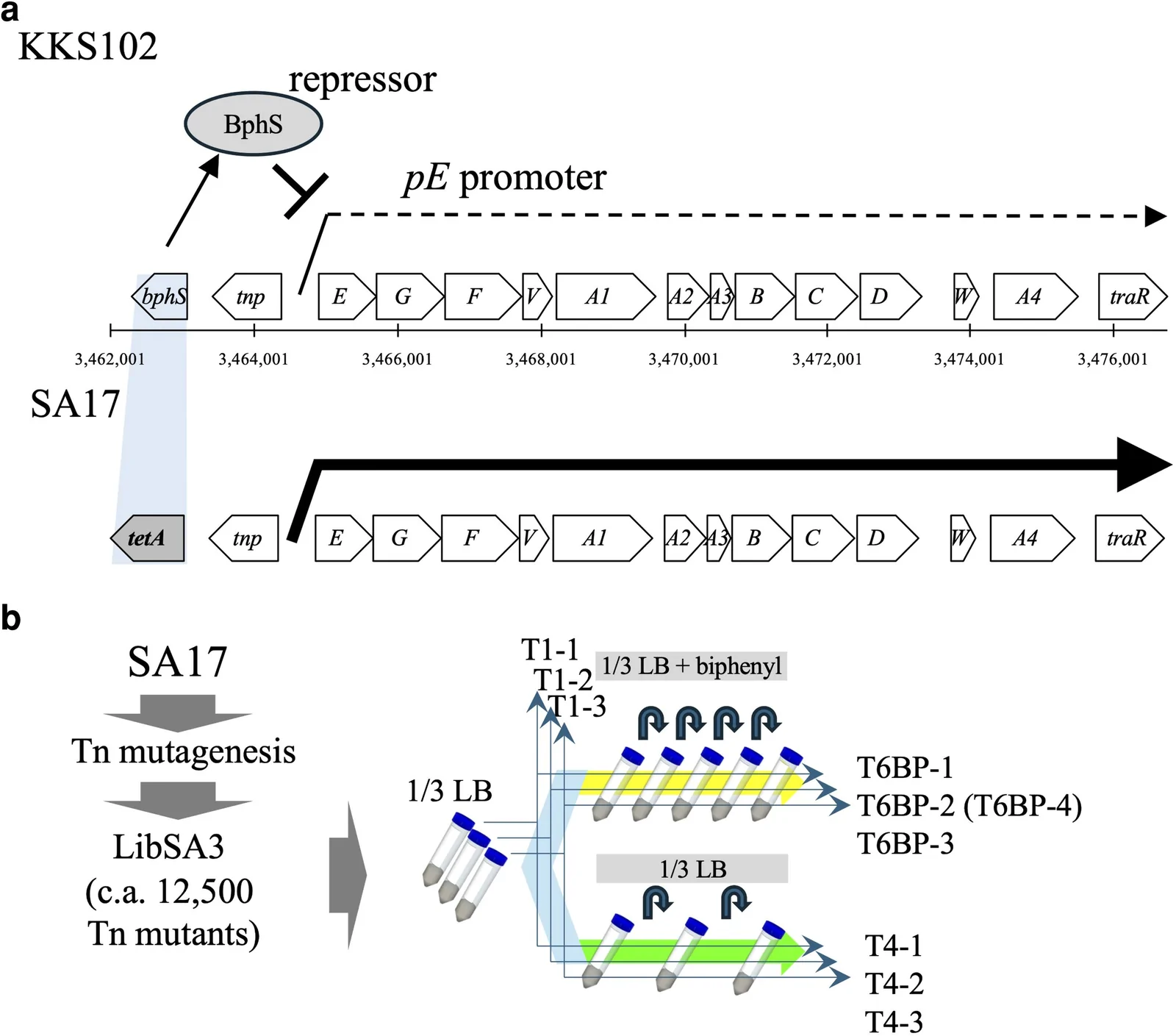
a) *Bph* operon in KKS102 and SA17, b) experimental scheme showing Tn mutant library cultivation (LibSA3). The freeze stock of LibSA3 was inoculated into fresh 1/3 LB liquid medium and incubated for 24 h (T1). A 1% volume of the T1 culture was then transferred into 1/3 LB medium without biphenyl and incubated for 24 h, resulting in the T2 culture. This process was repeated under the same conditions to produce the T3 and T4 cultures. Separately, a 1% volume of the T1 culture was also transferred into 1/3 LB medium without biphenyl, and biphenyl was added 8 h later, followed by an additional 27-h incubation, resulting in the T2BP culture. The T2BP culture was then inoculated at 1% into 1/3 LB medium pre-supplemented with biphenyl and incubated for 36 h to produce the T3BP culture. This process was continued under the same conditions to generate the T4BP, T5BP, and T6BP cultures. Both conditions involved triplicate passaging.

In this study, our objective was to utilize UMI-TnSeq in *Acidovorax* sp. KKS102 to identify genes that enhance fitness by either being highly expressed or disrupted when grown in the presence of biphenyl, to find mutations that increase fitness in the biphenyl-amended environment. We designed a series of Tn cassettes carrying different antibiotic resistance markers, with or without a promoter driving outward transcription. Using a Tn cassette with a promoter, we constructed a Tn-mutant library from a KKS102 derivative (strain SA17) lacking the *bphS* gene.^25^ SA17 expresses the *bph* operon due to the absence of the repressor, BphS, making it an ideal strain for identifying Tn mutations that increase fitness at levels other than transcription. The Tn-mutant library was repeatedly transferred and grown in the presence or absence of biphenyl, and the composition of each mutant in the library was analyzed using UMI-TnSeq to identify mutations that confer a fitness advantage in biphenyl-containing media. Additionally, we developed mathematical fomulas to estimate UMI-tag abundance in an NGS sequence library and introduced the Gini coefficient to evaluate population shifts toward fitter mutants.

## 2. Results

### 2.1. Design of Tn cassette

We designed 2 types of Tn cassette (Fig. 2). One of them carries a *tac* promoter that directs outward transcription. Both ends of the Tn cassettes contain 19-bp inverted repeats, known as ME sequences,^26^ which are recognized by Tn*5* transposase. The right end of the cassette harbors an I-CeuI site and a sequence for Illumina sequencing (P5 sequence, fixed index 2 sequence “TAGATCGC”, and read 1 primer annealing site). The annealing site overlaps with one of the ME sequences. For each type of cassette, we constructed a series of variants conferring resistance to trimethoprim, kanamycin, tetracycline, and gentamycin. See Figure S1 for the construction of plasmids carrying them.

**Fig. 2.**
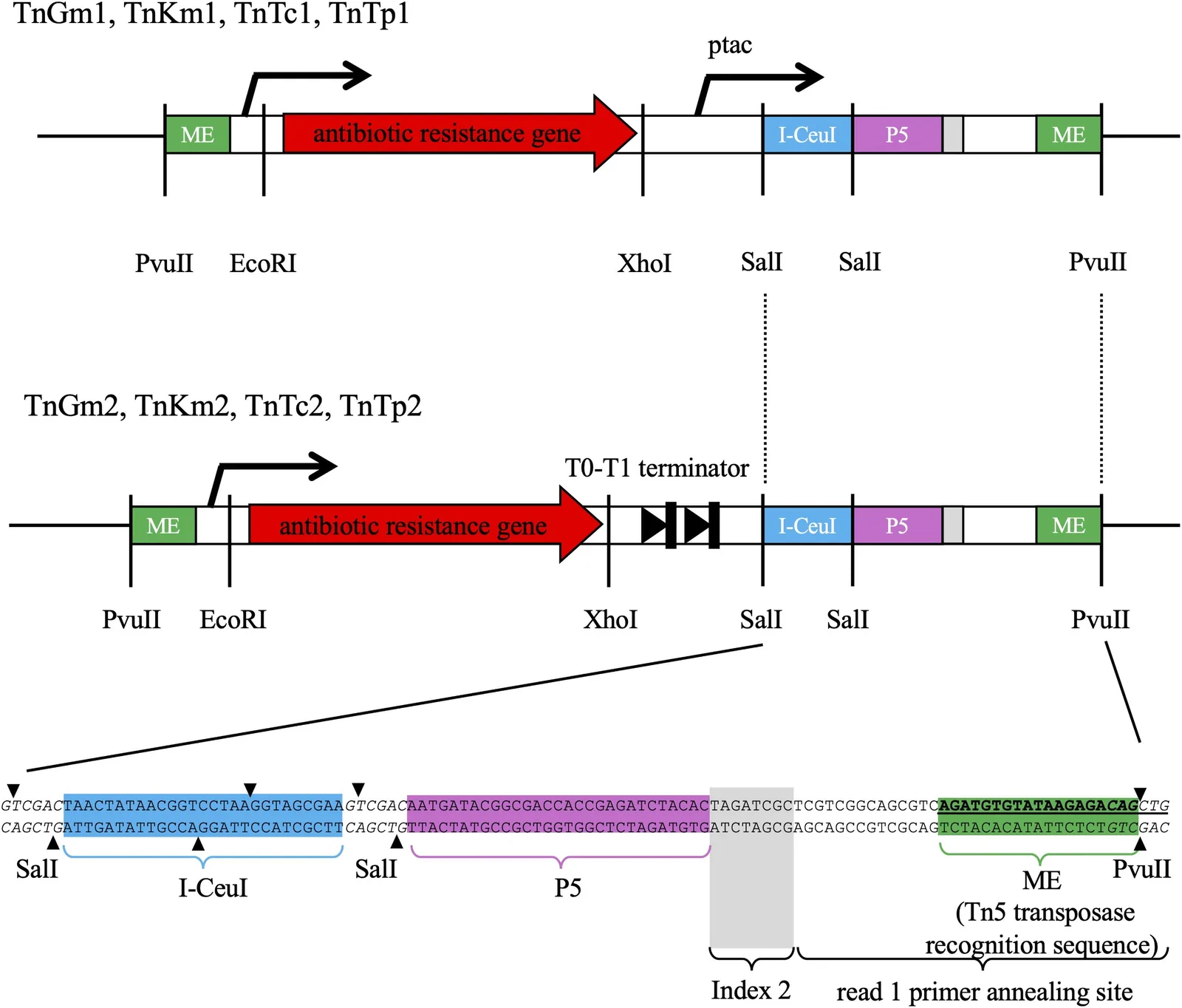
Transposon cassettes constructed in this study. We constructed 2 types of cassettes. One with outward *tac* promoter, and the other with T0–T1 terminator. The Tn-containing DNA was amplified by PCR using primers SA501 and SA502 and subsequently digested with PvuII to generate Tn-DNA. The nucleotide sequence between the SalI and PvuII sites is shown below. Restriction enzyme cleavage sites are indicated with triangles.

### 2.2. Tn mutant library construction

We introduced transposon mutations into the SA17 strain, which is derived from the KKS102 strain. SA17 has a disruption in the *bphS* gene, which encodes the transcriptional repressor of the *bph* operon, thereby overexpressing the *bph* operon. We used this overexpression strain to avoid selecting mutants that gain fitness through *bph* operon overexpression. Our goal was to gain insights into mutations that enhance fitness in the presence of biphenyl and to explore various factors involved in biphenyl degradation.

We introduced a transposon cassette (TnGm1) into the SA17 strain, generating a pool of approximately 12,500 mutants, which we named LibSA3. The relatively small library size was designed to ensure multiple UMI-tag counts per site while maintaining sufficient insertion density (∼1 per 200 bp in non-essential genes) and controlling sequencing costs. The transposon cassette used contains a gene conferring gentamicin resistance, an outwardly oriented promoter, sequences required for NGS analysis, etc. (see Fig. 2).

### 2.3. Growth of Tn mutant library

We treated the Tn mutant library, LibSA3, as shown in Fig. 1b. Initially, the library was grown once in 1/3 LB medium. Following this initial growth, the culture was split and further passaged in 1/3 LB medium either with or without biphenyl. The library was then serially passaged 3 times in 1/3 LB medium without biphenyl and 5 times in 1/3 LB medium with biphenyl. The number of passages was determined based on the results of a pilot experiment, aiming to make the Gini coefficients, described later, approximately equal. In other words, the Tn mutant library was passaged to ensure a similar degree of population shift toward fitter mutants across the 2 conditions. Cells after the first growth were collected in triplicate (Samples T1-1, T1-2, and T1-3), and cells from the serial passages were also collected (Samples T4-1, T4-2, and T4-3 for the medium without biphenyl, and Samples T6BP-1, T6BP-2, and T6BP-3 for the medium with biphenyl). Since Sample T6BP-2, grown with biphenyl, exhibited in subsequent analysis unexpectedly low diversity (see Table 1), we repeated the growth and cell collection, resulting in Sample T6BP-4.

**Table 1.** Data summary.

	T1-1	T1-2	T1-3	T4-1	T4-2	T4-3	T6BP-1	T6BP-2	T6BP-3	T6BP-4
Total pair count	6,290,188	3,623,445	3,692,617	5,367,795	4,287,406	3,059,383	5,462,087	4,007,759	6,395,507	41,876,931
Both-mapped pair count	5,178,201	3,028,120	3,070,758	4,452,215	3,518,032	2,546,015	4,499,504	3,277,193	4,911,015	34,657,048
Both-mapped-in-range pair count	4,901,395	2,960,271	2,976,981	4,339,459	3,313,550	2,455,164	4,337,815	3,113,951	4,779,910	31,898,672
Total insertion site detected	10,296	10,141	10,151	9,817	9,620	9,659	9,609	1,272	9,617	9,476
Total number of DNA molecule detected	1,754,510	1,044,421	1,155,757	1,561,247	1,169,896	1,130,511	1,529,534	1,307,573	1,187,558	3,279,735
Gini coefficient	0.4416	0.4476	0.453	0.7646	0.7836	0.7648	0.7966	0.9167	0.6508	0.9614
*N* estimate	2,163,366	1,273,551	1,460,507	1,940,780	1,430,907	1,533,599	1,886,930	1,700,955	1,312,755	3,075,160
Detection completeness value (%)	81.10	82.01	79.13	80.44	81.76	73.72	81.06	76.87	90.46	105.94

### 2.4. Preparation of NGS sequence library

The NGS sequence library was prepared as illustrated in Fig. 3. Briefly, total DNA extracted from the Tn mutant library was digested with I-CeuI to protect the region proximal to the Tn-end from sonication damage. After sonication and end-repair, a 3′ G-tailing reaction was performed to create an overhang of 3 to 5 Gs. A single-stranded DNA, referred to as GAO (guide adapter oligonucleotide), was then incorporated using the CIS reaction, in which a complementary strand was synthesized. The GAO contained N9D2 (a degenerate sequence where “N” is A, T, G, or C, and “‘D” is A, G, or T), which, together with 30 bases of adjacent genomic sequence, served as the UMI for molecular identification. Finally, a PCR reaction was carried out to prepare the NGS sequence library.

**Fig. 3.**
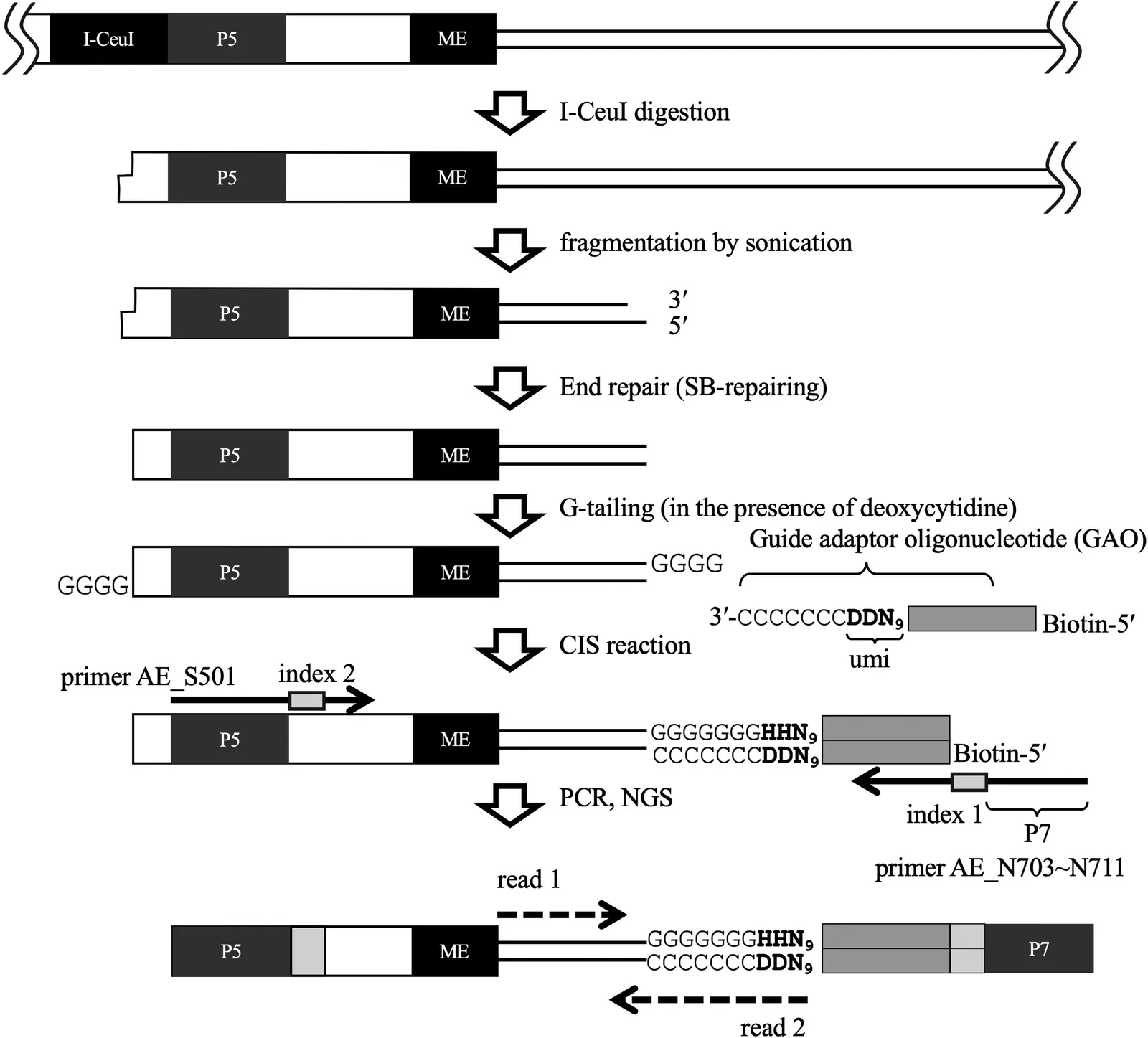
NGS sequence library preparation incorporating UMI tags. Total DNA was digested with I-CeuI and subsequently fragmented by sonication. Following Scrap-and-Build repairing, DNA termini were G-tailed using the strong tailing activity of reverse transcriptase in the presence of the G-tailing enhancer, deoxycytidine. To the G-tail, guide adaptor oligonucleotides (GAOs) containing UMI sequences and a stretch of Cs at their 3′ termini were applied and incorporated via a CIS reaction mediated by reverse transcriptase. The GAOs were designed with a 5′ biotin to inhibit the 3′ tailing reaction on the opposing newly synthesized strand, preventing recursive GAO incorporation and the formation of complex reaction products.^17^ Finally, PCR was performed to prepare the NGS sequence library for sequencing.

### 2.5. NGS read acquisition and data analysis

Illumina sequencing was performed to obtain NGS reads. The read pairs stored in 2 files were processed using a computational tool named GenomeTagMatcher to identify Tn locations in the genome and to count the number of UMI tags associated with each location. Further analysis was conducted to calculate the Gini coefficient, the N-estimate, and the Detection Completeness Value, as discussed below. The results for 10 datasets are presented in Table 1.

### 2.6. Calculation of the Gini coefficient to assess mutant library composition

We employed the Gini coefficient to assess the composition of the Tn mutant library (Table 1 and Figure S2). The Gini coefficient indicates the extent to which the library's composition is skewed toward certain strains. As mutants with advantageous mutations increase and those with disadvantageous mutations decrease in each environment, the Gini coefficient is expected to rise. This makes it a valuable measure for determining whether the selection period was sufficiently long to identify genes relevant to fitness in that environment. In our preparative experiments, we observed that the Gini coefficient increased much faster in cultures grown without biphenyl compared to those grown with biphenyl. Based on these observations, we decided to passage the cultures 6 times with biphenyl and 4 times without biphenyl.

## 3. Estimation of NGS library diversity and read acquisition sufficiency

In this section, we introduce a mathematical method to estimate how sufficiently an NGS library has been sequenced, under the assumption that the NGS sequencing library composition is uniform. Our model does not aim to evaluate the bias introduced during PCR amplification or library preparation, but rather to assess the completeness of read acquisition based on a random sampling model.

The following formula, *f*(*x*), calculates the expected number of tags observed exactly *x* times, where *m* represents the total number of NGS reads, and *n* represents the total number of distinct DNA (UMI) species in the sequence library. Here, mCx is the binomial coefficient, ie the number of combinations of *x* elements chosen from *m*.


$$f(x){=}_{m}C_{x}\times {\left(\frac{1}{n}\right)}^{x}\times \;{\left(\frac{n-1}{n}\right)}^{m-x}\times n$$

Our objective is to estimate *n* based on *m* and the observed *Px*, which represents the number of tag species observed *x* times (*x* = 1, 2, 3, …). The value of *n* that minimizes the sum of squared differences calculated using the formula *g*(*n*) below is the estimated value of *n* (referred to as *N_est_*), which can be determined programmatically.


$$g(n)\;=\;\underset{x=1}{\sum }\;\{\;f(x)-P_{x}{\}}^{2}$$

The ratio of the actual number of detected DNA species to the *N_est_* is defined as the Detection Completeness Value (DCV). *N_est_* serves as a measure of the quality or diversity of the NGS sequence library. If the efficiency of UMI adapter attachment is low, a small *N* value would be expected. On the other hand, the DCV serves as an indicator of NGS read sufficiency; a low DCV suggests the need for additional NGS read acquisition to achieve sufficient detection.

### 3.1. Evaluation of nest and DCV by rarefaction analysis

To evaluate the usefulness of the *N_est_* and completeness value, we conducted rarefaction analysis (Fig. 4 and Table S2) using the largest read set we obtained in this study (T6BP-4, 41876931 reads). The rarefied NGS read set with different dilution rates were used for tag-counting, and subsequent estimation of N and calculation of the DCV. In this analysis tag-counting data was binned based on the insert distance, for a better estimate (see our python script nest.py). The *N_est_* value calculated using total reads was 3.08 M, which is close to the value of 2.86 M obtained with a dilution rate of 1% and stayed stable at higher dilution rates, suggesting that Nest is a stable indicator of the NGS sequence library. In this case, DCV is 105.9% and further NGS reads seems unnecessary.

**Fig. 4.**
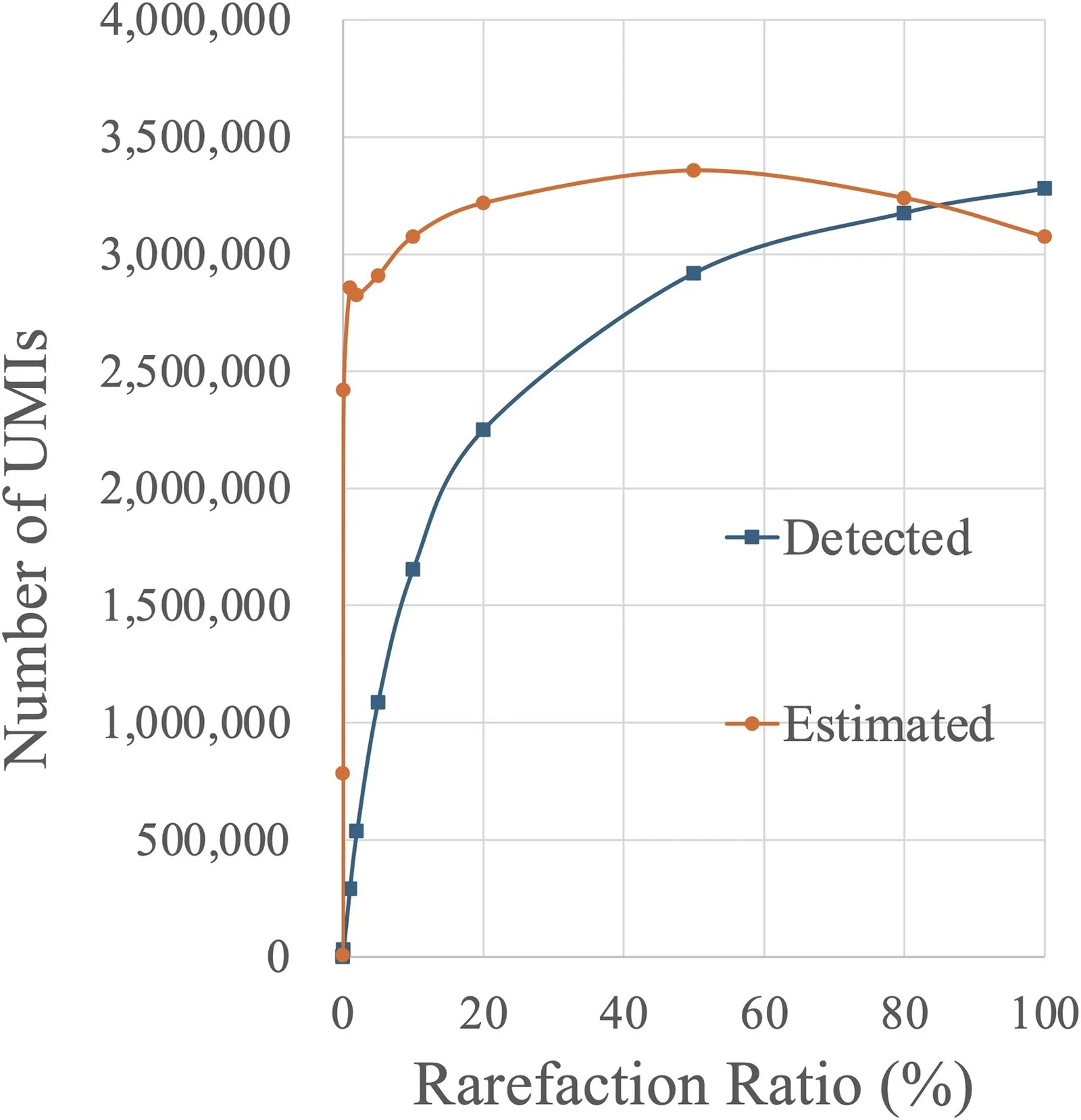
Accuracy of total UMI estimation with limited NGS reads. Rarefaction analysis was performed using the dataset with the highest read count (41.9 M), containing 3.2 M unique UMIs. Subsets of reads at the indicated rarefaction ratios (percentage of total reads) were analyzed to determine the total UMIs present in the dataset (squares) and to estimate the total number of UMIs in the NGS sequencing library (circles). Estimations were performed using the Python script nest.py.

### 3.2. Mutations that increased the relative abundance in media containing biphenyl

Although T6BP-2 was included when defining the union set of 12,257 Tn insertion sites, it was excluded from subsequent statistical analyses because it showed markedly low insertion-site diversity (Table 1). The relative abundance of each mutant strain in the library was calculated by dividing the number of unique UMI types detected for each mutant by the total number of unique UMI types detected in a given sample and then multiplying by one million (UUPM; unique UMI per million). UUPM data for each insertion site across 3 experimental conditions, along with statistical comparisons and significance values (2-sample Welch's *t* test), are provided in Table S3. Comparison of T4 and T6BP resulted in fifteen insertion sites where the UUPM in T6BP was more than twice that in T4, with a *P*-value below 0.05. With our primary goal of identifying mutations that enhance fitness in the presence of biphenyl, we excluded 4 sites where UUPM was either zero or nearly zero in the T4 sample and below 10 in the T6BP sample, listing the remaining eleven sites in Table 2 (These sites were reproducibly identified when T6BP-4 data was 10% down sampled, Table S4). Among the eleven insertion sites, the highest increase in UUPM was observed at insertion site ID 11623, which showed a 227.9-fold increase in T6BP compared to T4. The effect of Tn insertion is likely attributed to the upregulation of nearby genes. Transcription driven by the promoter embedded in the Tn cassette is believed to have led to the overexpression of several genes, including 7 genes from C380_22770 to C380_22740 (Fig. 5a).

**Fig. 5.**
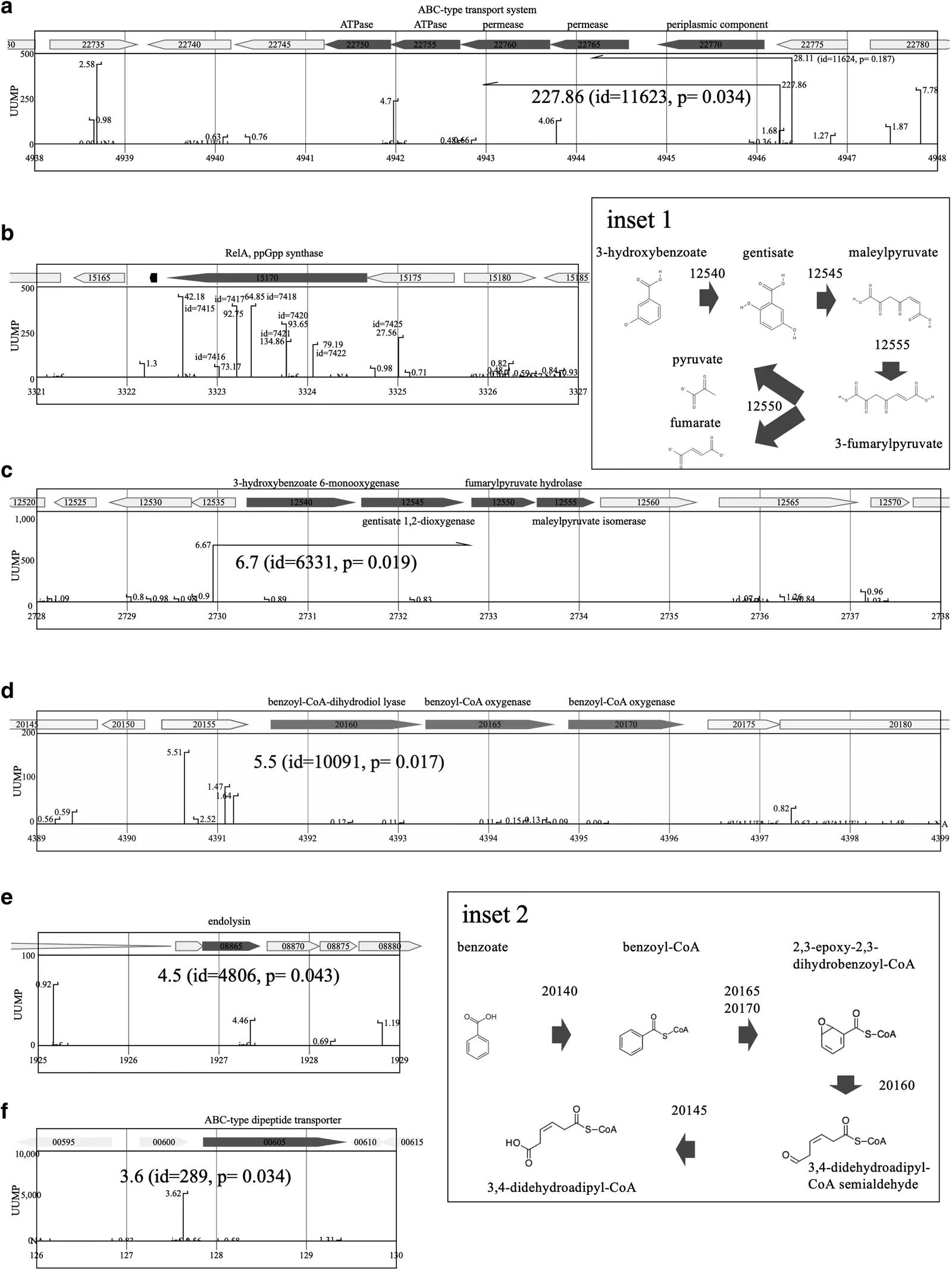
Genomic loci related to increased abundance upon insertion of the promoter-carrying tn cassette. The Tn insertion sites identified in this study that contributed to increased abundance are shown. The *x*-axis represents genomic location, while the *y*-axis represents UUPM. Thin arrows in the middle panel indicate the direction of outward transcription from the Tn cassette. Genes predicted to be involved in fitness increase are highlighted in dark gray and labeled with their predicted gene products. The black gene represents tRNA gene. For key insertion sites, the insertion site ID and corresponding *P*-value are indicated. Additionally, the insets illustrate the degradation pathways of 3-hydroxybenzoate (inset 1) and benzoate (inset 2) encoded in genomic loci shown in panels (c) and (d), respectively. The numbers indicate KKS102 locus_ tags (C380_XXXXX). The figure was generated based on genome maps drawn using the GeneDraw function in GenomeMatcher software.^27^

**Table 2.** Tn mutants increased in the presence of BP.

Tn insertion site id	Genomic location	Transcription direction	T6_BP UUPM (mean)	T6_BP/T4 fold change	*P*-value (2-sample Welch's *t*-test)	T6_BP/T1 fold change	*P*-value (2-sample Welch's *t*-test)	Tn insertion effect	Note	T6_BP/T1 fold change^a^	*P*-value^a^	FDR^a^	T6_BP/T4 fold change^a^	*P*-value^a^	FDR^a^
11623	4,946,256	−1	326.7	227.9	0.034	20.6	0.037	Upregulation of C380_22770, 22765, 22760, 22755, 22750, 22745, and 22740	Overexpression of an ABC-type benzoate transporter system. CDD search of C380_22770 identified a conserved domain, cd20013 for benzoate transport. The system is similar to branched-chain amino acid transport system	146.8	1.92 × 10⁻⁵	4.75 × 10⁻⁴	641.8	6.80 × 10⁻⁶	4.85 × 10⁻⁴
7421	3,323,766	1	197.5	134.8	0.043	6.6	0.058	Insertion within C380_15170 (*relA* gene)	Inactivation of *relA* gene	36.9	6.63 × 10⁻⁵	9.12 × 10⁻⁴	343.7	1.13 × 10⁻⁵	6.49 × 10⁻⁴
7420	3,323,764	−1	294.3	93.6	0.036	6.6	0.048	Insertion within C380_15170 (*relA* gene)	Inactivation of *relA* gene	39.4	6.81 × 10⁻⁵	9.21 × 10⁻⁴	248.1	8.76 × 10⁻⁶	5.81 × 10⁻⁴
7422	3,324,061	1	180.3	79.2	0.048	5.8	0.066	Insertion within C380_15170 (*relA* gene)	Inactivation of *relA* gene	31.1	1.40 × 10⁻⁴	1.38 × 10⁻^3^	189.9	3.25 × 10⁻⁵	1.01 × 10⁻^3^
7416	3,323,021	−1	58.0	73.2	0.039	2.3	0.097	Insertion within C380_15170 (*relA* gene)	Inactivation of *relA* gene	11.9	2.07 × 10⁻^3^	9.46 × 10⁻^3^	156.9	8.06 × 10⁻⁴	7.85 × 10⁻^3^
7418	3,323,376	1	394.3	64.8	0.042	6.0	0.058	Insertion within C380_15170 (*relA* gene)	Inactivation of *relA* gene	33.4	4.06 × 10⁻⁴	2.81 × 10⁻^3^	159.6	5.90 × 10⁻⁵	1.32 × 10⁻^3^
7415	3,322,618	−1	445.7	42.2	0.022	6.8	0.029	Insertion within C380_15170 (*relA* gene)	Inactivation of *relA* gene	42.5	1.41 × 10⁻⁴	1.39 × 10⁻^3^	115.1	3.77 × 10⁻⁵	1.07 × 10⁻^3^
7425	3,325,009	1	219.2	27.6	0.037	4.7	0.054	Insertion within C380_15175	Possible polar effect on *relA* expression	26.9	1.42 × 10⁻⁴	1.39 × 10⁻^3^	69.6	4.23 × 10⁻⁵	1.12 × 10⁻^3^
6331	2,729,950	1	627.8	6.7	0.019	3.6	0.027	Insertion within C380_12535. Upregulation of C380_12540, 12545, 12550, 12555, 12560, and 12565.	Overexpression of genes for 3-hydoxybenzoate catbolism	32.4	1.20 × 10⁻^3^	6.12 × 10⁻^3^	25.9	1.74 × 10⁻^3^	1.40 × 10⁻^2^
10091	4,390,634	1	158.4	5.5	0.017	1.7	0.072	Upregulation of C380_20160, 20165, and 20170	Overexpression of genes for benzoate catbolism via benzoyl-CoA	14.9	2.14 × 10⁻^3^	9.72 × 10⁻^3^	21.2	1.21 × 10⁻^3^	1.06 × 10⁻^2^
4806	1,927,347	1	27.9	4.5	0.043	0.7	0.100	Insertion within C380_08865	Inactivation of a gene for endolysin	4.3	4.51 × 10⁻^2^	1.08 × 10⁻^1^	11.9	1.31 × 10⁻^2^	5.84 × 10⁻^2^
289	127,630	1	5286.0	3.6	0.052	9.5	0.035	Upregulation of C380_00605	Overexpression of gene for ABC-type dipeptide transporter	57.6	5.35 × 10⁻⁴	3.42 × 10⁻^3^	9.3	1.20 × 10⁻^2^	5.49 × 10⁻^2^

^a^NB analysis was performed with edgeR using raw UMI counts and quasi-likelihood *F*-tests based on negative binomial generalized linear models. T6BP-2 was excluded. Full data are provided in Table S3.

These genes are predicted to encode an ABC-type benzoate transporter system. Benzoate is a metabolic intermediate produced during biphenyl degradation. It is likely that some of the benzoate generated is protonated, forming lipophilic benzoic acid, which can diffuse across the cell membrane and be released into the medium as benzoate. The overexpression of the benzoate transporter system is considered essential for the reuptake of extracellular benzoate. This may explain why the relative abundance of mutants with high expression of this system was enhanced.

Seven of the identified insertion sites were located within the same genomic locus (Fig. 5b). Among them, 6 were inserted within the *relA* gene, while the remaining site (site ID 7425) was inserted in its immediate vicinity. Insertion into site ID 7425was presumed to have disrupted *relA* function due to a polar effect. The mutant strains carrying these insertions showed a 27.4- to 136-fold increase in UUPM.

*relA* is known to be involved in the synthesis of ppGpp, a key regulator of the stringent response. The disruption of *relA* likely prevented the activation of the stringent response, allowing cells to remain active at the onset of energy depletion, when sufficient energy for growth was still present, thereby increasing their abundance.

Insertion of the Tn element at site ID 6331 (Fig. 5c) appears to have led to the overexpression of genes involved in the metabolism of 3-hydroxybenzoate. Although the exact pathway of 3-hydroxybenzoate formation in the presence of biphenyl remains unclear, it is predicted that the pathway converting 3-hydroxybenzoate to gentisate, and subsequently to pyruvate and fumarate, is upregulated in the Tn insertion mutant.

Insertion at site ID 10091 (Fig. 5d) seems to lead to the overexpression of the benzoyl-CoA metabolic pathway, encoded by *C380_20160* to *C380_20170*. The enzyme gene responsible for converting benzoate to benzoyl-CoA is located nearby at *C380_20140* although *C380_20140* is unlikely to be upregulated by Tn insertion due to its orientation. These findings suggest that a portion of the benzoate derived from biphenyl is degraded via the benzoyl-CoA pathway.

Additionally, insertion at site ID 4806 disrupted *C380_08865* (Fig. 5e), which is annotated as encoding a putative phage-related endolysin. The disruption of this gene may reduce the risk of cell lysis due to endolysin activity, potentially contributing to increased fitness under these conditions.

A similar analysis comparing T1 and T6BP under the same conditions identified 6 mutations. Among these, 5 were already detected in the T4 versus T6BP analysis described above. The remaining mutation occurred at insertion site ID 289 (Fig. 5f), which is predicted to enhance the transcriptional activation of *C380_00605*, a gene encoding a dipeptide transporter. This mutation showed a 2.63-fold increase in UUPM when comparing T1 and T4, suggesting that it may be involved in the utilization of dipeptides present in 1/3 LB medium.

## 4. Discussion

### 4.1. Mutations giving increased abundance in medium containing biphenyl

In this study, “fitness” refers to the ability of a mutant to increase its relative abundance within the pooled mutant population during serial cultivation under the specified condition. Here, we identified several Tn insertion mutations in the KKS102 genome that enhanced relative abundance in the presence of biphenyl. Notably, many of these mutations were associated with genes related to benzoate uptake and metabolism, which have been less studied in the context of biphenyl degradation in KKS102.

The Tn insertion at site ID 6284 induced the expression of the 3-hydroxybenzoate metabolic pathway, although its precise contribution to biphenyl metabolism remains unclear. One possibility is that the metabolic pathway has broad substrate specificity and can also utilize benzoate. Another possibility is that 2,3-dihydroxybenzoate is produced as a minor byproduct during biphenyl mineralization. Biphenyl is typically metabolized through the sequential activities of BphA and BphB, introducing hydroxyl groups at the 2,3 positions of one aromatic ring, followed by ring cleavage by BphC and hydrolysis by BphD to produce benzoate and 2-hydroxypenata-2,4-dienoate.^28^ However, an alternative route may exist in which both aromatic rings undergo dihydroxylation before ring cleavage, leading to the formation of 2,3-dihydroxybenzoate. Given that BphA can utilize various chlorinated biphenyls as substrates,^24^ it is plausible that a biphenyl with one dihydroxylated ring could serve as a substrate for BphA, resulting in the production of a compound with dihydroxyl groups on both rings. The same possibility applies to BphB and BphC. Compared to benzoate, 2,3-dihydroxybenzoate is more likely to be processed by the series of enzymes encoded near site ID 6331 (Fig. 5c). This could explain why the high expression of genes surrounding site ID 6331 contributed to increased relative abundance.

Another intriguing mutation was the disruption of the *relA* gene, which encodes an enzyme involved in ppGpp synthesis.^29^ ppGpp is a well-known regulator of the stringent response, inhibiting growth under starvation or other stressful conditions.^29^ Why was the *relA* mutant enriched in our experiment? One possible explanation is that KKS102 may activate the stringent response unnecessarily early even when some growth substrates remain available. In a serial transfer experiment like ours, where biphenyl serves as the primary carbon source, KKS102 cells that enter the stringent response too early slow down their growth, while *relA* mutants could continue proliferating, leading to a higher cell count over successive passages.

While the precise functions of these genes require further experimental validation, our findings demonstrate that these mutations increased relative abundance. An advantage of the UMI-TnSeq method is that it allows the identification of mutants with increased abundance even when gene functions are not fully understood. Furthermore, the observed increased abundance provides clues for inferring gene functions, highlighting the utility of this approach in uncovering genetic determinants of fitness in different bacteria under various environments.

### 4.2. Advantages of the UMI-ligation method used in this study

In this study, we employed a novel UMI-ligation method, which enables to ligate a single-stranded DNA fragment containing UMI efficiently. Compared to approaches that rely on restriction enzyme MmeI, our method enables retrieval of sufficiently long adjacent genomic sequences, allowing for accurate mapping of insertion sites and identification of their genomic origins. A known technical limitation of previous methods utilizing MmeI is the short length of the obtained genomic sequence, which can hinder unique mapping. For example, in the 4.6 Mb genome of *Escherichia coli* B str. REL606 (accession number NC_012967), there are 4,043,156 unique 14 bp sequences. This means that a randomly chosen 14 bp sequence cannot be uniquely mapped with a probability of 12.7%. Previous reports using mariner/MmeI-based TnSeq and INSeq systems have further noted that, after trimming transposon- and adaptor-derived sequences, only approximately 13 bp of each read remain available for mapping to the host genome.^30^ Under these conditions, the probability that a read cannot be uniquely assigned to a single genomic location becomes even higher (in the case of REL606, 29.4%), particularly in large or repeat-rich genomes. In the genome sequence of KKS102 (accession no. CP003872), 52.6% of 13-bp sequences and 30.6% of 14-bp sequences are not unique. In contrast, our method^17^ enables the acquisition of much longer genomic sequences, thereby eliminating this limitation.

### 4.3. Uniqueness of the UMIs used in this study

In this study, we used the initial 41-base sequence of read 2 as a UMI, consisting of an 11-base sequence derived from the synthetic N9D2 oligonucleotide and 30 bases derived from the adjacent genomic sequence. The N9D2 sequence alone has a theoretical diversity of only 4^9^ × 3^2^ = 2,359,296 possible combinations. However, the additional use of 30 bases of genomic sequence greatly increases the diversity of the UMIs, making the probability that 2 independent DNA molecules share the same UMI negligible. In the N9D2 sequence, D represents any nucleotide except C (A, G, or T). The terminal DD was designed to distinguish the random sequence from the consecutive Cs immediately downstream.

In our data analyses, we allowed up to 2 errors in the 11 bp tag sequences. This approach was chosen because allowing no errors would lead to an artificially inflated number of unique tags, caused by random sequencing errors in the reads. Allowing a small number of errors helps maintain accuracy while accounting for sequencing imperfections.

### 4.4. Evaluation of the effect of UMI-Tag use on quantitative accuracy

To assess how the use of UMI tags improves the accuracy of quantification, we analyzed dataset T6BP-4, which yielded the highest number of reads among our samples. For each Tn insertion site, we calculated the ratio between the total number of reads (read count) and the number of unique UMI sequences associated with that site (UMI count). For example, if 100 reads were grouped into 20 UMI tags at a given insertion site, the ratio would be 5. This ratio reflects the degree of read duplication at each insertion site, and variability in this ratio among the total sites indicates the effectiveness of correcting for duplicated reads using UMI information.

The results are shown in Table 3. Among the 9,476 insertion sites detected in T6BP-4, the average read-to-UMI ratio was 9.12, with a standard deviation of 4.15. We also binned the data based on UMI count and calculated the mean and standard deviation for each bin. In the bin with the lowest UMI count (≤10), the average ratio was 9.23 with a standard deviation of 5.12. This indicates a large variation (55.49% of the mean), suggesting that without UMI correction, reliable comparison between 2 samples would be quite difficult. Indeed, the maximum value in this bin was 47.5, which is over 5 times the average.

**Table 3.** Variability in read-to-UMI count ratio across UMI count bins in T6_BP.

	Total	UMI count bins						
		1–10	11–20	21–50	51–100	101–200	201–500	Over 500
Number of Tn sites	9,476	2,636	2,608	1,845	473	196	267	266
Average ratio (read count/UMI count)	9.12	9.23	9.12	9.17	9.08	9.29	9.19	9.34
Variability (standard deviation)	4.15	5.12	2.73	2.10	1.72	1.45	1.32	1.23
Max ratio	55.00	47.50	21.07	23.51	13.17	12.37	15.44	16.05
Minimum ratio	1.000	1.000	1.000	1.077	1.270	1.235	1.325	1.379
%CV (variability/average ratio)	45.49	55.49	29.96	22.92	18.99	15.64	14.33	13.21

In contrast, the bin with UMI count ≥500 showed an average ratio of 9.34 and a standard deviation of only 1.23. The fact that smaller UMI count bins exhibit larger standard deviations is particularly important because the majority of Tn mutants fall into these lower-count bins. These results highlight that the use of UMI tags is especially critical for accurately quantifying Tn mutants with low abundance.

### 4.5. Gini coefficient as a measure of population shift toward fitter strains

In this study, we used the Gini coefficient to measure the extent to which the composition of Tn mutant strains in the library shifted toward fitter strains in response to environmental conditions. The composition of the Tn mutant library is expected to change as fitter mutants increase in relative abundance while less fit mutants decrease, resulting in a more monopolized state dominated by fewer strains. This shift is reflected by an increase in the Gini coefficient. The Gini coefficient serves as a single numerical value that captures the initial diversity of the library and the degree of shift in mutant composition after exposure to specific conditions, making it a useful metric for understanding the extent of selection toward fitter mutants and for comparing shifts across different experiments. Consequently, the Gini coefficient is anticipated to be a crucial indicator in UMI-TnSeq analysis.

### 4.6. Enhancing UMI-based analyses through N-estimate and DCV

In this study, we developed a method for calculating the *N*-estimate. Given *N* unique UMI tags and performing *m* random draws with replacement, the expected number of tags detected 1 to *k* times can be determined mathematically. Our approach identifies the value of *N* that minimizes the sum of squared errors between the observed number of tags detected 1 to *k* times and the mathematical expectations. This *N*-estimate serves as an indicator of the diversity of the constructed sequencing library. If *N* is very low, it implies that the sequencing library lacks sufficient diversity, suggesting poor library quality.

The *N*-estimate assumes that UMI-tagged molecular species are uniformly represented in the NGS sequencing library. A poor fit between the observed and theoretically expected frequency distributions indicates deviation from this assumption, reflecting uneven representation of UMI-tagged molecular species in the sequencing library.

Without UMI tags, it is challenging to discern whether a limited number of detected Tn mutants is due to insufficient sequencing library quality or a genuine shift toward fitter mutants. The *N*-estimate directly informs us about the extent of UMI tag detection, enabling the calculation of DCVs. If the *N*-estimate is sufficiently high and the DCV is low, it suggests that acquiring additional reads could further enhance data quality. Thus, *N*-estimate and DCV provide crucial numerical indicators for assessing sequencing library quality (in terms of UMI tag richness) and determining whether sufficient reads have been obtained.

These 2 metrics are broadly applicable to UMI-based research and are expected to contribute significantly to future studies employing UMI technology. A Python script for calculating these values is provided in the Supplementary material. In this Python script, the data are binned based on the length from the Tn end to the genomic junction, with bins created in increments of 50 base pairs (eg 1 to 50, 51 to 100, 101 to 150, and so on). The *N*-estimate is calculated separately for each bin, and the final *N*-estimate is obtained by summing the values from all bins.

### 4.7. Evaluating the reliability of *N*-estimation through rarefaction analysis

We performed rarefaction analysis to investigate the number of reads required for approximately accurate *N*-estimation. For this purpose, we used the dataset with the highest number of reads (41.9 M), from which a total of 3.28 M unique UMI tags were ultimately detected. The rarefaction analysis demonstrated that with only 1% of the data (0.42 M reads), it was possible to estimate 2.86 M UMIs, corresponding to 87% of the total UMIs detected in the full dataset. This indicates that even when the number of reads is insufficient due to technical or experimental issues, the approximate number of UMI tags present in the sequencing library can still be inferred.

However, while these results highlight the utility of the *N*-estimate in assessing UMI diversity, cautions must be taken when dealing with datasets with extremely low read numbers. In such cases, the *N*-estimate may be significantly inaccurate, potentially leading to misinterpretations. Therefore, ensuring a minimum threshold of read depth is critical when employing *N*-estimation in practical applications. Further investigations to establish this threshold across diverse experimental setups would enhance the robustness and reliability of this metric.

### 4.8. GIIF: the third concept for elucidating microbial lives in natural environments

Various research methods have been developed to extend the study of bacteria from laboratory media to understanding how they live in their natural environments. Techniques such as IVET (in vivo expression technology) and STM (signature-tagged mutagenesis) have been developed for this purpose. IVET is used to identify genes that are selectively expressed in specific environments, revealing pathways and functions that are upregulated in response to environmental conditions.^31–36^ STM, on the other hand, is designed to identify genes that are essential for survival in specific environments by searching for mutant-specific tags that are depleted by exposure to a particular environment.^37–39^

Besides these methodologies, transposon-based techniques have been utilized to identify genes that, when disrupted or transcriptionally induced by transposon-embedded outward promoters, enhance bacterial fitness. Notable examples include TraDIS^5^ and study by Coward et al.^40^ To encapsulate this experimental approach, we propose the term ‘Gene Identification based on Increased Fitness’ (GIIF). GIIF is a method to identify genes that upon disruption or transcriptional activation enhance the fitness of microbes. The aim of GIIF is more direct than identifying in vivo specific gene functions (IVET) or fitness-decreasing mutations (STM) and then testing if overexpression of the related gene increases fitness. Importantly, GIIF complements IVET and STM as a third concept focused on elucidating how bacteria adapt and survive in complex environments. The identified possible fitness-increasing mutations could be combined cumulatively to further enhance fitness. The concept of GIIF would be valuable for applied microbiology studies, as it enables the direct identification and utilization of fitness-enhancing mutations to optimize microbial performance in research and application areas such as environmental remediation, wastewater treatment, probiotic functionality, and agricultural biotechnology.

## 5. Materials and methods

### 5.1. Culture conditions

The bacterial strains and plasmids used in this study are listed in Table 4. *Escherichia coli* strains were grown at 37 °C in Luria–Bertani (LB) broth, which contained 10 g of tryptone, 5 g of yeast extract, and 5 g of NaCl per liter. Strains derived from the KKS102 strain, including the Tn mutant library, were grown at 30 °C in 1/3 Luria–Bertani (1/3LB) broth, which contained 3.33 g of tryptone, 1.67 g of yeast extract, and 5 g of NaCl per liter. When solid media were required, agar was added at a concentration of 1.5% (w/v). When necessary, antibiotics were used at the following concentrations: for KKS102-derived strains, gentamicin (Gm) was used at either 5 µg/mL or 2 µg/mL, and tetracycline (Tc) at either 5 µg/mL or 2 µg/mL. For *E. coli* strains, gentamicin was used at 10 µg/mL, tetracycline at 10 µg/mL, and kanamycin (Km) at 10 µg/mL.

**Table 4.** Strains and plasmids.

Strain or plasmid	Relevant characteristics	Source or references
Strains		
*Acidovorax* sp. KKS102	PCBs/biphenyl degrader, carries ICEKKS102Tn*4677*.	^44^
SA10	KKS102 derivative, in which the *bphS* gene was replaced by the tetracycline resistance gene.	^19^
SA17	KKS-SA10 derivative, in which *traR* gene was replaced by chloramphenicol resistance gene.	This study
Library		
LibSA3	SA17-derived Tn mutant library: The TnGm1 cassette, a transposon with an outward-facing promoter, was used to generate the library, which consists of approximately 12,500 Tn mutant clones.	
Plasmids		
pGiTp	Synthetic construct based on pUC57 (accession #Y14837), carry trimethoprim resistance gene between 2 ME sequences.	^14^
pUC18-miniTn7T-LAC	Used to amplify T0–T1 terminator sequences	AY599234
pTnTp1	A plasmid carrying a Tn cassette with an outward-facing promoter and a trimethoprim resistance gene. See Fig. 2.	This study
pTnTp2	A plasmid carrying a Tn cassette with T0–T1 terminator and a trimethoprim resistance gene. See Fig. 2.	This study
pTnTc1	A plasmid carrying a Tn cassette with an outward-facing promoter and a tetracycline resistance gene. See Fig. 2.	This study
pTnTc2	A plasmid carrying a Tn cassette with T0–T1 terminator and a tetracycline resistance gene. See Fig. 2.	This study
pTnGm1	A plasmid carrying a Tn cassette with an outward-facing promoter and a gentamycin resistance gene. See Fig. 2.	This study
pTnGm2	A plasmid carrying a Tn cassette with T0–T1 terminator and a gentamycin resistance gene. See Fig. 2.	This study
pTnKm1	A plasmid carrying a Tn cassette with an outward-facing promoter and a kanamycin resistance gene. See Fig. 2.	This study
pTnKm2	A plasmid carrying a Tn cassette with T0–T1 terminator and a kanamycin resistance gene. See Fig. 2.	This study

### 5.2. Construction of tn cassette carrying plasmids

In this study, we constructed 2 types of transposon cassette (Tn cassette) containing plasmids, as shown in Figure S1, based on the plasmid pGiTp (a synthetic plasmid, referenced in.^14^) These 2 types are distinguishable by their naming convention: those with an outward-facing *tac* promoter have names ending in ‘1’, while those with T0-T1 transcription terminators instead of a promoter have names ending in ‘2’. We created 4 variants for each type, each carrying a different antibiotic resistance gene. These genes include gentamicin (Gm) resistance, kanamycin (Km) resistance, tetracycline (Tc) resistance, and trimethoprim (Tp) resistance genes, derived from pEX18Gm, pTnMod-OKm, pTnMod-OTc, and pTnMod-OTp', respectively.^41^

The original pGiTp plasmid contains an outward-facing promoter. An I-CeuI site was introduced into pGiTp, resulting in the construction of pTnTp1. Additionally, the promoter sequence of pGiTp was replaced with a DNA fragment containing the T0-T1 terminator from pUC18-mini-Tn7T-LAC^42^ and an I-CeuI site, creating pTnTp2. Both pTnTp1 and pTnTp2 were subsequently digested with EcoRI and XhoI to replace the Tp resistance gene with Gm, Km, and Tc resistance genes, resulting in the construction of pTnGm1, pTnGm2, pTnKm1, pTnKm2, pTnTc1, and pTnTc2.

### 5.3. Tn mutant library construction

The Tn cassettes were prepared by amplifying DNA fragments containing Tn cassettes using primers SA501 and SA502, and then digesting them with the restriction enzyme PvuII to generate ME sequences^26^ at the both ends of the DNA. The digestion gives a 5′-terminus phosphate, which is expected to enhance the efficiency of transposome DNA introduction, according to the manual for the Ez-Tn*5* transposase (Epicentre, an Illumina Company, San Diego, CA, USA).

The prepared Tn cassette DNA was mixed with transposase to create a transposome solution. Strain SA17 was cultured, washed with Milli-Q water, mixed with the transposome solution, and subjected to electroporation. The conditions for electroporation were 25 µF, 200 Ω, and 1.5 kV, using a 1 mm cuvette. Selection was conducted using 1/3 LB medium containing 10 ppm gentamicin. The resulting colonies were counted, amounting to approximately 12,500, which were then pooled together to create the transposon library LibSA3.

### 5.4. Cultivation of Tn mutant library in the presence or absence of biphenyl

The Tn mutant library was cultured in 5 mL of 1/3 LB medium at 30 °C using a Bio-Photo Recorder TVS062CA (ADVANTEC Co.). When biphenyl was added, approximately 5 mg of biphenyl powder (Wako Pure Chemical Industries) was introduced. For the series cultured with biphenyl, the initial addition of biphenyl was conducted 8 h after inoculating the cells into 1/3 LB medium. This approach was taken because biphenyl exhibits toxicity to the KKS102 strain,^43^ particularly when cells from a pre-culture grown in the absence of biphenyl are inoculated into medium containing biphenyl, resulting in severe growth inhibition. In contrast, cultures incubated for 36 h in the presence of biphenyl retained residual biphenyl, and subsequent inoculations into biphenyl-containing medium did not show growth inhibition. After incubation, the cells were harvested, and total DNA was extracted.

### 5.5. DNA extraction and sonication, SB-repairing

Cells were harvested, and genomic DNA was extracted using the NucleoSpin Tissue kit (Macherey-Nagel, headquartered in Düren, Germany). Depending on the sample, either 100 ng or 200 ng of genomic DNA was used. The DNA was digested using I-CeuI (New England Biolabs, headquartered in Ipswich, MA, USA) and then fragmented by sonication using the Misonix Microson Ultrasonic Cell Disruptor XL processor (Misonix, Inc., Farmingdale, NY, USA) for 5 s at intensity level 4. Fragment termini were repaired by the SB-repairing method (see Reference,^16^) employing a mixture of Exonuclease III and T4 DNA Polymerase in the presence of dNTPs for 60 min at 37 °C. The repaired fragments were then purified using a column purification kit.

### 5.6. G-tailing reaction

The G-tailing reaction was performed in the presence of deoxycytidine as a specific enhancer. A 10 µL reaction mixture was prepared as follows: 4.75 µL of DNA solution, 2 µL of 5× reaction buffer, 0.4 µL of 100 mM dGTP, 0.2 µL of 100 mM DTT, 2 µL of 1 M deoxycytidine, 0.4 µL of 100 mM MnCl2, and 0.25 µL of Moloney Murine Leukemia Virus Reverse Transcriptase (MMLV-RT, Nippon Gene Co., Ltd., Tokyo, Japan). The mixture was incubated for 1 h at 37 °C, and the DNA was subsequently purified by using a column purification kit.

### 5.7. CIS reaction

The CIS reaction was initiated by combining 2 solutions. Solution A (3.5 µL) contained 2 µL of 5× buffer, 0.2 µL of 100 mM DTT, 1 µL of 2.5 mM each of dNTPs, and 0.3 µL of MMLV-RT. Solution B (6.5 µL) contained 5 µL DNA solution and 1.5 µL of 2 µM guide adaptor oligonucleotide SA718. Solution A was placed at the bottom of a tube, while Solution B was stored in the lid of the tube. The tube was pre-warmed at 37 °C, and the solutions were mixed by centrifugation and tapping, and then incubated at 37 °C for 30 min. Excess guide adaptor oligonucleotide was digested by adding 1 µL of Exonuclease I (Takara Bio, headquartered in Shiga Prefecture, Japan) and incubating for 15 min at 37 °C. Exonuclease I was inactivated by incubating at 80 °C for 15 min.

### 5.8. NGS sequence library preparation

The CIS reaction product was used as a template for PCR amplification. The primers used were listed in Table S1.

### 5.9. NGS data analysis

NGS sequencing was performed using a commercial service provided by GENEWIZ (Azenta Life Sciences, South Plainfield, NJ, USA). Paired-end 150 bp sequencing data were obtained using the Illumina platform. The raw sequencing data generated in this study have been deposited in the NCBI Sequence Read Archive (SRA) under the accession numbers SRX27826959 to SRX27826968, associated with BioProject PRJNA1228261.

The following outlines the NGS data analysis implemented in GenomeTagMatcher, an Objective-C based command line tool, which is available at https://github.com/ohtsuboy/UMI-TnSeq-tools.

**1. K-mer Indexing**: DNA sequences from a multi-FASTA file are indexed using 21-mers to allow for efficient searching.
**2. Mapping of Read Pairs**:
  1. The initial k-mer is extracted from read1, and the k-mer following the 18nt-UMI tag segment at the beginning of read2 is also extracted.
  2. Each pair is mapped to a genomic location where the facing distance between the reads is the shortest. Only pairs with a distance between 50 and 800 bp are retained for analysis.
**3. Binning of Read Pairs**: Read pairs are binned based on the location of read1, which corresponds to the Tn cassette location. Further binning is performed based on the location and initial 41 bases of read2. These 41 bases include an 11-mer UMI tag and 30 bases from the genomic sequence.
**4. Tag Counting**: For each Tn location, read2 data is sorted by the distance from the Tn cassette. The initial 41-base sequence is used to identify and combine reads originating from a single DNA molecule, allowing for up to 2 mismatches to avoid overcounting.
**5. Data Export**: Two files are generated:
  1. A summary file reporting the number of DNA molecules detected at each Tn insertion site.
  2. A detailed file providing information for each DNA molecule, including the initial 41-base tag sequence, distance from the Tn end, and occurrence count. This file serves as the input for the Python script (Nest.py).

### 5.10. Calculation of the Gini coefficient

To calculate the Gini coefficient, we first ranked the detected UMI tags for each Tn mutant in ascending order based on their abundance. We then calculated the cumulative proportion of each mutant's abundance to generate the Lorenz curve. The Gini coefficient was determined as the ratio of the area between the Lorenz curve and the line of equality (representing an equal distribution) to the area of the triangle formed by the line of equality. When all mutants are present in similar proportions, the Gini coefficient should be close to 0. In contrast, when a limited number of mutants dominate, the Gini coefficient approaches 1. Thus, the Gini coefficient serves as an indicator of the selection toward fitter mutants. The Python script (gini.py) used for this analysis is available at https://github.com/ohtsuboy/UMI-TnSeq-tools.

### 5.11. Estimation of N using Python script

The estimation of the number of distinct DNA molecules (N) in the Tn mutant library was performed using a Python script. This script reads NGS data output from the GenomeTagMatcher application, processes it by binning the data based on the distance from the Tn end, and determines the frequency distribution of observed tags within these bins. The script calculates the sum of observed tags (“read2 occurrence”) for each bin and uses an optimization approach to find the value of n that minimizes the sum of squared differences between the expected and observed distributions. In cases where no duplicate UMIs are observed in a bin, a calculation method is employed, where *N* is estimated as the smallest value where the probability of observing no duplicates exceeds that of observing one duplicate.

The input file is a tab-separated values file with a header starting on the seventh line. The script processes data based on the maximum occurrence limit (max occurrence), defaulting to 20 (rows with “read2 occurrence” value exceeding the “max occurrence” are ignored). The output includes the estimated number of distinct DNA molecules (*N*) for each bin, as well as the detection completeness value (DCV), indicating the sufficiency of the NGS read acquisition. The Python script (nest.py) used for this analysis is available upon request.

## Supplementary Material

dsag017_Supplementary_Data
