# Supplementary material for: Estimating UMI-Tag count: UMI-TnSeq protocol for identifying fitness-relevant genes in the PCB degrader Acidovorax sp. KKS102
Source: DNA Res. 2026 Oct 30;33(6):dsag017. doi: 10.1093/dnares/dsag017 (PMC13721135; doi:10.1093/dnares/dsag017)
Supplement: Supplementary file 1 [file dsag017_supplementary_data.zip › FigureS1.pdf]

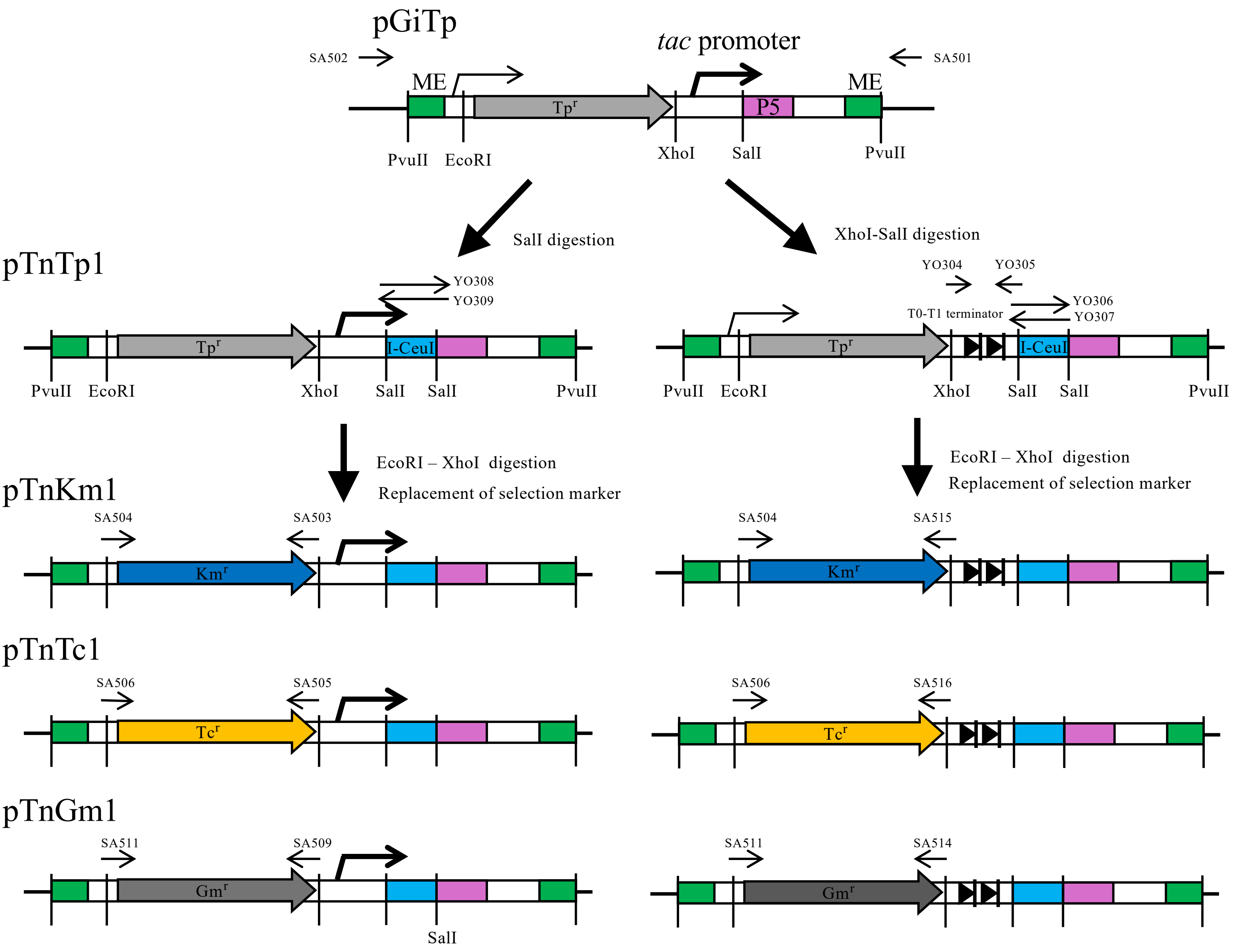

Fig. S1 Construction of Two Series of Tn Cassettes with Different Antibiotic Resistance Markers

The plasmid pGiTp [13] was synthesized using GeneWiz's plasmid synthesis service. It is based on the pUC57 vector (accession number Y14837), with a Tn cassette cloned into its multiple cloning site, flanked by two Mosaic End sequences [24]. The Tn cassette contains a trimethoprim resistance gene, an outward-facing *tac* promoter, a P5 sequence for Illumina sequencing, and several restriction enzyme sites. To create pTnTp1, an I-CeuI site was added to pGiTp. To construct pTnTp2, the *tac* promoter of pGiTp was replaced with a transcription terminator and an I-CeuI site. The antibiotic resistance gene in pTnTp1 was replaced as indicated in the figure to generate pTnKm1, pTnTc1, and pTnGm1. Similarly, the resistance gene in pTnTp2 was replaced to construct pTnKm2, pTnTc2, and pTnGm2. The positions, orientations, and names of the relevant oligonucleotides are shown in the figure. The sequences of the oligonucleotides are listed in Table S1.
