# Supplementary material for: Estimating UMI-Tag count: UMI-TnSeq protocol for identifying fitness-relevant genes in the PCB degrader Acidovorax sp. KKS102
Source: DNA Res. 2026 Oct 30;33(6):dsag017. doi: 10.1093/dnares/dsag017 (PMC13721135; doi:10.1093/dnares/dsag017)
Supplement: Supplementary file 1 [file dsag017_supplementary_data.zip › FigureS2.pdf]

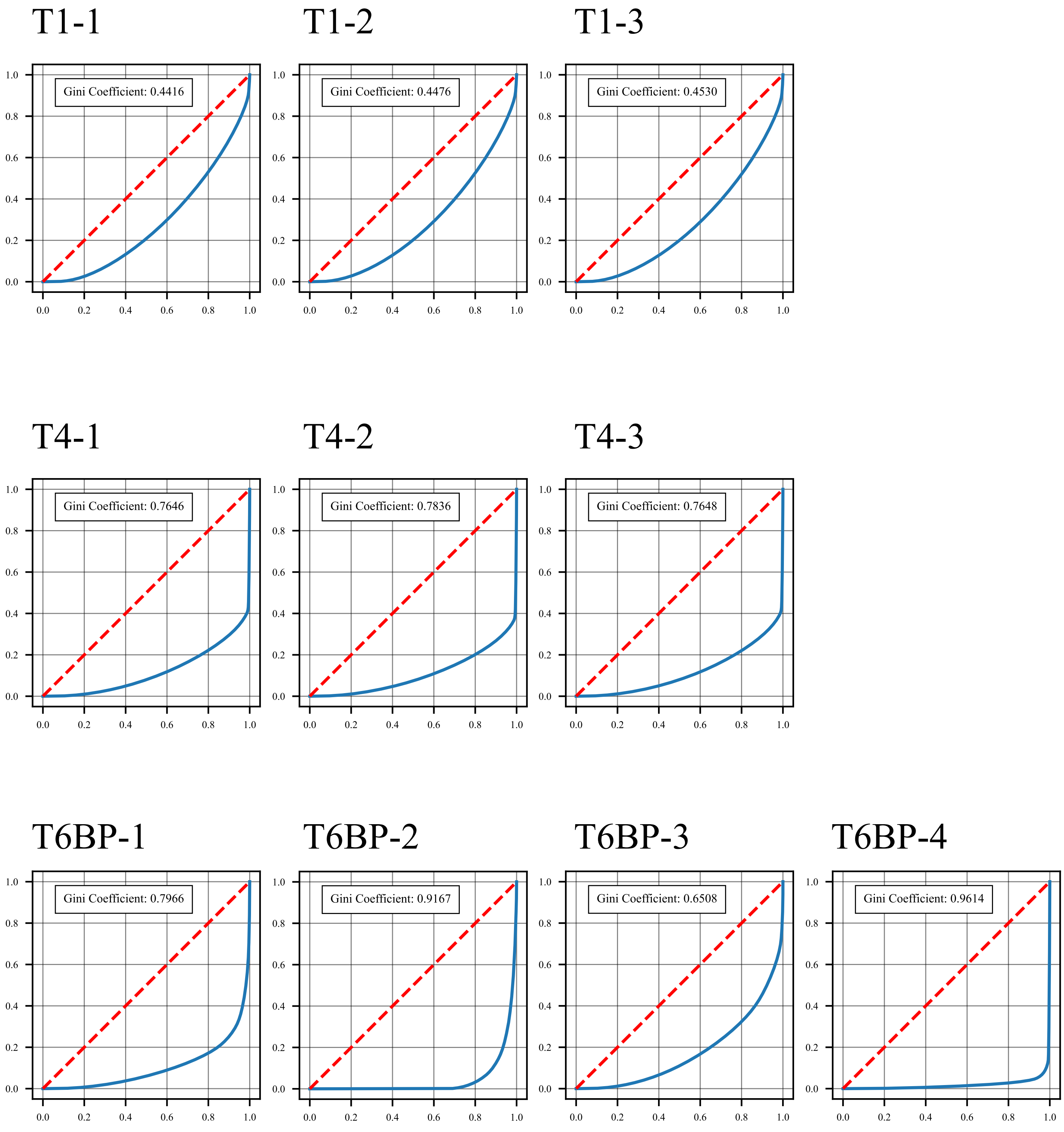

Fig. S2 Gini Coefficient Analysis of Tn Mutant Libraries Before and After Cultivation in the Presence or Absence of Biphenyl

The tag counts of each mutant were sorted in ascending order to construct a Lorenz curve (blue line), from which the Gini coefficient was calculated. The Gini coefficient quantifies the inequality of tag distribution among mutants and serves as an indicator of the extent to which fitter mutants dominate the population. It is defined as the ratio of the area between the Lorenz curve and the line of equality (red line) to the total area under the line of equality. Graphs were generated using the Python script gini.py.
