# Supplementary material for: Estimating UMI-Tag count: UMI-TnSeq protocol for identifying fitness-relevant genes in the PCB degrader Acidovorax sp. KKS102
Source: DNA Res. 2026 Oct 30;33(6):dsag017. doi: 10.1093/dnares/dsag017 (PMC13721135; doi:10.1093/dnares/dsag017)
Supplement: Supplementary file 1 [file dsag017_supplementary_data.zip › TableS1.pdf]

Table S1. Oligonucleotides and primers used in this study

name	sequence	note
YO304	TGTGGCCTAACTCGAGGACTCCTGTTGATAGATCC	Amplification of T0-T1 terminator using pUC18-miniTn7T-LAC as a template. Used in combination with YO305.
YO305	GGCGGATTTGTCCTACTCAGG	See YO304
YO306	CCTGAGTAGGACAAATCCGCCTAACTATAACGGTCCTAAGGTAGCGAAGTCGACAATGATACGG	Annealed with YO306 to create DNA fragment containing I-CeuI site. Used to construct pTnTp1.
YO307	CCGTATCATTGTCGACTTCGCTACCTTAGGACCGTTATAGTTAGGCGGATTTGTCCTACTCAGG	See YO306
YO308	TATAATGTGTGGAAGTCGACTAACTATAACGGTCCTAAGGTAGCGAAGTCGACAATGATACGGCGACCAC	Annealed with YO309 to create DNA fragment containing I-CeuI site. Used to construct pTnTp2.
YO309	GTGGTCGCCGTATCATTGTCGACTTCGCTACCTTAGGACCGTTATAGTTAGTCGACTTCCACACATTATA	See YO308
SA501	CATTCAGGCTGCGCAACTGTTG	Amplification of the Tn cassete containing DNA fragment
SA502	GCAGCGAGTCAGTGAGCGAG	Amplification of the Tn cassete containing DNA fragment
SA503	AGAATATTTG*CTCGAG* TTAGAAAAACTCATCGAGCATCA	Amplification of kanamycin resistance gene for the construction of pTnKm1. Used in combination with SA504
SA504	TAATGCAAGT*GAATTC* AAAGCCACGTTGTGTCTCAA	Amplification of kanamycin resistance gene for the construction of pTnKm1 and pTnKm2
SA505	AGAATATTT*GCTCGAG* TCAGGTCGAGGTGGCCCGG	Amplification of tetracycline resistance gene for the construction of pTnTc1. Used in combination with SA506
SA506	TAATGCAAGT*GAATTC* ATGTTTGACAGCTTATCATCCGATTAG	Amplification of tetracycline resistance gene for the construction of pTnTc1 and pTnTc2
SA509	AGAATATTTG*CTCGAG* TTAGGTGGCGGTACTTGGGTCG	Amplification of gentamycin resistance gene for the construction of pTnGm1. Used in combination with SA511
SA511	TAATGCAAGTGAATTCGTATGCGCTCACGCAACTGG	Amplification of gentamycin resistance gene for the construction of pTnGm1 and pTnGm2
SA514	TCAACAGGAGTC*CTCGAG* TTAGGTGGCGGTACTTGGGTCG	Amplification of gentamycin resistance gene for the construction of pTnGm2. Used in combination with SA511
SA515	TCAACAGGAGTC*CTCGAG* TTAGAAAAACTCATCGAGCATCA	Amplification of kanamycin resistance gene for the construction of pTnKm2. Used in combination with SA504
SA516	TCAACAGGAGTC*CTCGAG* TCAGGTCGAGGTGGCCCGG	Amplification of tetracycline resistance gene for the construction of pTnTc2. Used in combination with SA506
SA718	biotin-GTGACTGGAGTTCAGACGTGTGCTCTTCCGATCTNNNNNNNNNDDCCCCCCC	Used for CIS reaction
AE_S501	AATGATACGGCGACCACCGAGATCTACACTAGATCGCTCGTCGGCAGCGTCAGAT	P5 containing primer for NGS sequence library preparation, with the index of TAGATCGC
AE_N703	CAAGCAGAAGACGGCATACGAGATTTCTGCCTGTGACTGGAGTTCAGACGTGT	P7 containing primer for NGS sequence library preparation, with the index of TTCTGCCT
AE_N704	CAAGCAGAAGACGGCATACGAGATGCTCAGGAGTGACTGGAGTTCAGACGTGT	Same as AE_N703, except that index is GCTCAGGA
AE_N705	CAAGCAGAAGACGGCATACGAGATAGGAGTCCGTGACTGGAGTTCAGACGTGT	Same as AE_N703, except that index is AGGAGTCC
AE_N706	CAAGCAGAAGACGGCATACGAGATCATGCCTAGTGACTGGAGTTCAGACGTGT	Same as AE_N703, except that index is CATGCCTA
AE_N707	CAAGCAGAAGACGGCATACGAGATGTAGAGAGGTGACTGGAGTTCAGACGTGT	Same as AE_N703, except that index is GTAGAGAG
AE_N708	CAAGCAGAAGACGGCATACGAGATCCTCTCTGGTGACTGGAGTTCAGACGTGT	Same as AE_N703, except that index is CCTCTCTG
AE_N709	CAAGCAGAAGACGGCATACGAGATAGCGTAGCGTGACTGGAGTTCAGACGTGT	Same as AE_N703, except that index is AGCGTAGC
AE_N710	CAAGCAGAAGACGGCATACGAGATCAGCCTCGGTGACTGGAGTTCAGACGTGT	Same as AE_N703, except that index is CAGCCTCG
AE_N711	CAAGCAGAAGACGGCATACGAGATTGCCTCTTGTGACTGGAGTTCAGACGTGT	Same as AE_N703, except that index is TGCCTCTT
