# Supplementary material for: Estimating UMI-Tag count: UMI-TnSeq protocol for identifying fitness-relevant genes in the PCB degrader Acidovorax sp. KKS102
Source: DNA Res. 2026 Oct 30;33(6):dsag017. doi: 10.1093/dnares/dsag017 (PMC13721135; doi:10.1093/dnares/dsag017)
Supplement: Supplementary file 1 [file dsag017_supplementary_data.zip › TableS2.pdf]

Table S2 N-estimate and DCV values obtained in a rarefaction analysis of reads obtained from T6BP-4

Number of Reads	Rarefaction Ratio(%)	UMIs detected	N-estimate	DCV (%)	N-estimate / Full-data Detected Count*
419	0.001	322	8,099	3.98	0.00
4,188	0.01	3,162	784,521	0.4	0.24
41,877	0.1	31,624	2,420,424	1.31	0.74
418,769	1	291,495	2,855,723	10.21	0.87
837,539	2	537,115	2,825,293	19.01	0.86
2,093,847	5	1,087,362	2,908,306	37.39	0.89
4,187,693	10	1,654,498	3,075,621	53.79	0.94
8,375,386	20	2,249,954	3,218,279	69.91	0.98
20,938,466	50	2,918,718	3,357,492	86.9	1.02
33,501,545	80	3,176,478	3,239,737	97.82	0.99
41,876,931	100	3,279,735	3,075,160	105.94	0.94

*The “N-estimate / Full-data Detected Count” value was calculated by dividing the N-estimate obtained at each rarefaction level by the total number of UMI-tagged molecular species detected using the complete dataset (3,268,443). Thus, this value indicates how closely the N-estimate obtained from a subsampled dataset approaches the number of molecular species detected with the full dataset.
