# Supplementary material for: Estimating UMI-Tag count: UMI-TnSeq protocol for identifying fitness-relevant genes in the PCB degrader Acidovorax sp. KKS102
Source: DNA Res. 2026 Oct 30;33(6):dsag017. doi: 10.1093/dnares/dsag017 (PMC13721135; doi:10.1093/dnares/dsag017)
Supplement: Supplementary file 1 [file dsag017_supplementary_data.zip › TableS4.docx]

Table S4 Analysis of Tn insertion sites using 10% downsampled T6BP-4 reads

| Tn insertion site id | Genomic location | Transcription Direction | T6_BP UUPM  (mean) | T6_BP/T4 fold change | p-value  (two-sample Welch’s t-test) | T6_BP/T1 fold change | p-value  (two-sample Welch’s t-test) | T6_BP/T1 fold change* | p-value* | FDR* | T6_BP/T4 fold change* | p-value* | FDR* |
| --- | --- | --- | --- | --- | --- | --- | --- | --- | --- | --- | --- | --- | --- |
| 11623 | 4,946,256 | -1 | 310.3 | 216.5 | 0.048 | 19.5 | 0.052 | 126.3253179 | 4.89E-06 | 1.80E-04 | 550.271577 | 1.63E-06 | 1.58E-04 |
| 7421 | 3,323,766 | 1 | 199.0 | 136.0 | 0.040 | 6.6 | 0.053 | 37.32317983 | 8.15E-05 | 8.85E-04 | 348.01363 | 1.58E-05 | 5.51E-04 |
| 7420 | 3,323,764 | -1 | 286.2 | 91.1 | 0.048 | 6.4 | 0.064 | 35.60480386 | 2.93E-05 | 4.91E-04 | 225.346307 | 3.27E-06 | 2.44E-04 |
| 7422 | 3,324,061 | 1 | 178.3 | 78.3 | 0.053 | 5.8 | 0.074 | 29.06074937 | 1.93E-04 | 1.56E-03 | 178.032892 | 5.45E-05 | 1.09E-03 |
| 7416 | 3,323,021 | -1 | 55.7 | 70.2 | 0.056 | 2.2 | 0.149 | 8.975547844 | 1.35E-03 | 6.55E-03 | 118.850157 | 3.60E-04 | 4.21E-03 |
| 7418 | 3,323,376 | 1 | 398.3 | 65.5 | 0.038 | 6.0 | 0.051 | 34.53530357 | 2.68E-04 | 1.99E-03 | 165.191999 | 3.57E-05 | 8.50E-04 |
| 7415 | 3,322,618 | -1 | 436.9 | 41.4 | 0.030 | 6.6 | 0.039 | 39.89123822 | 6.81E-05 | 7.92E-04 | 108.008422 | 1.59E-05 | 5.51E-04 |
| 7425 | 3,325,009 | 1 | 218.0 | 27.4 | 0.039 | 4.7 | 0.058 | 26.11849748 | 1.22E-04 | 1.13E-03 | 67.60228 | 3.66E-05 | 8.57E-04 |
| 6331 | 2,729,950 | 1 | 624.3 | 6.6 | 0.018 | 3.5 | 0.025 | 32.11109595 | 8.81E-04 | 4.73E-03 | 25.7054079 | 1.31E-03 | 1.10E-02 |
| 10091 | 4,390,634 | 1 | 153.8 | 5.3 | 0.012 | 1.6 | 0.057 | 13.76564209 | 2.29E-03 | 9.91E-03 | 19.5758087 | 1.29E-03 | 1.09E-02 |
| 4806 | 1,927,347 | 1 | 25.3 | 4.0 | 0.072 | 0.6 | 0.094 | 2.687006851 | 7.78E-02 | 1.68E-01 | 7.44359726 | 1.11E-02 | 5.23E-02 |
| 289 | 127,630 | 1 | 5200.1 | 3.6 | 0.063 | 9.4 | 0.042 | 54.79605021 | 4.81E-04 | 3.03E-03 | 8.88271067 | 1.27E-02 | 5.78E-02 |
| *The analysis was performed for all Tn insertion sites using the same pipeline as in Table 3, except that T6BP-4 was replaced by data generated from 10% downsampled T6BP-4 reads. This table shows only the 12 candidate sites listed in Table 3. | | | | | | | | | | | | |  |
